# An ACE2-IgG4 Fc Fusion Protein Demonstrates Strong Binding to All Tested SARS-CoV-2 Variants and Reduced Lung Inflammation in Animal Models of SARS-CoV-2 and Influenza

**DOI:** 10.20411/pai.v7i1.491

**Published:** 2022-08-23

**Authors:** Emmanuel Y. Merigeon, Dong Yang, Elizabeth A. Ihms, Leda C. Bassit, Elizabeth A. Fitzpatrick, Colleen B. Jonsson, Raymond F. Schinazi, David S. Block, Henrik S. Olsen

**Affiliations:** 1 Gliknik Inc., Baltimore, MD; 2 Regional Biocontainment Laboratory, University of Tennessee Health Science Center, Memphis, TN; 3 Carlson College of Veterinary Medicine, Oregon State University, Corvallis, OR; 4 Emory University School of Medicine and Children's Healthcare of Atlanta, Department of Pediatrics, Atlanta, GA; 5 Dept. of Microbiology, Immunology and Biochemistry, University of Tennessee Health Science Center, Memphis, TN

**Keywords:** ACE2, COVID-19, SARS-CoV-2 antiviral agents, anti-inflammatory

## Abstract

**Background:**

The continued emergence of SARS-CoV-2 variants has caused concern that a constantly evolving virus will escape vaccines and antibody therapies. New approaches are needed.

**Methods:**

We created and manufactured an ACE2 extracellular domain (ECD) fragment Fc fusion drug candidate, G921, and engineered the compound for enhanced delivery of drug to peripheral tissues by minimizing the size of the ACE2 ECD and by incorporating an Fc domain to enhance transcytosis. G921 was assessed for binding, neutralization, *in vivo* anti-inflammatory effect, and pharmacokinetic profile.

**Results:**

G921 was expressed as an IgG4 Fc fusion protein presenting two ACE2 domains to ACE2 ligands while avoiding risk of infection via antibody-dependent enhancement. G921 strongly binds to the SARS-CoV-2 Wuhan-Hu-1 spike protein and demonstrates further diminished off rate to the spike protein from each of the currently identified variants of concern. G921 demonstrates ACE2 enzymatic activity comparable to positive control and binding to the neonatal Fc receptor (FcRn) without binding to low affinity Fc-gamma receptors (FcγRs). G921 is effective in a concentration-dependent manner in a focus reduction neutralization assay with EC_50_=16.3±4.2 µg/mL without cytotoxicity in Vero E6 cells when tested at 200 µg/mL in an MTS cell proliferation assay. G921 demonstrates statistically significant reduction of lung inflammation in relevant models of both SARS-CoV-2 and influenza. The pharmacokinetic profile demonstrated dose-dependent exposure with a multi-day half-life in monkeys and rats.

**Conclusion:**

G921 data are consistent with both antiviral and anti-inflammatory modes of action. G921 is a novel approach for the prevention and treatment of COVID-19 and possible other diseases characterized by deficiency of ACE2.

## INTRODUCTION

Currently available COVID-19 therapeutics are insufficient to address emerging severe acute respiratory syndrome coronavirus-2 (SARS-CoV-2) variants of concern (VOCs), particularly for the unvaccinated population. Here, we describe the preclinical evaluation of G921, a homodimeric angiotensin-converting enzyme 2 (ACE2) IgG4 Fc fusion protein therapeutic that incorporates extracellular domain (ECD) fragments of two ACE2 moieties with the Fc portion of human IgG4 and which demonstrates bifunctional activity in both viral neutralization and anti-inflammatory ACE2 replenishment. G921 may be useful in the prevention and treatment of COVID-19 caused by SARS-CoV-2 VOCs.

Cell entry of SARS-CoV-2 is mediated through the enzyme ACE2, which is naturally in a dimeric state as an integral membrane protein in the plasma membrane [1, 2]. Dimeric ACE2 can accommodate two SARS-CoV-2 S protein trimers simultaneously, potentially permitting clustering between dimeric ACE2 and trimeric S proteins, which may contribute to invagination of the membrane and endocytosis of the viral particle [3]. ACE2 is abundantly present in human epithelial cells of the lung and enterocytes of the small intestine as well as in endothelial cells of the arterial and venous vessels [4]. Interaction between the receptor-binding domain in the viral spike protein and the ACE2 initiates the cell entry and endocytosis process [5]. Cell surface ACE2 expression levels and enzymatic function may be significantly lowered due to the endocytosis of the spike:ACE2 complex [6]. The viral cell entry process thus can create a potential deficiency of enzymatically active membrane-bound ACE2 [7].

ACE2 functions as a cell surface enzyme that hydrolyzes about a dozen peptides at the C-terminus [8], including the pro-inflammatory angiotensin II (Ang II) to anti-inflammatory angiotensin 1-7 (Ang-(1-7)) and inflammatory des-Arg^9^-bradykinin to its breakdown products. ACE2 plays a central role in the renin-angiotensin system by modulating Ang II-Angiotensin II receptor type 1 (AT1R) signaling, which mediates inflammatory reactions including vasoconstriction, inflammation, fibrosis, arrhythmias, apoptosis, and thrombosis. ACE2 cleavage of Ang II generates Ang-(1-7), which binds and activates the Mas receptor [9]. Ang-(1-7) Mas signaling mediates anti-inflammatory responses including vasodilation, anti-fibrosis, anti-arrhythmia, anti-apoptosis, and anti-thrombotic responses and thus has an opposite effect to the Ang II-AT1R inflammatory pathway. While the Ang II-AT1 receptor and the Mas receptor pathways are normally kept in balance, it has been proposed that SARS-CoV-2-induced inflammation can be viewed as the result of a virus-induced ACE2 deficiency, resulting in an increased ratio of Ang II to Ang-(1-7) favoring an inflammatory response [10, 11].

As the COVID-19 pandemic has progressed worldwide, several viral VOCs with increased infectivity and mortality have emerged [12]. Increased ACE2 binding affinity to viral spike protein of all current WHO VOCs has been demonstrated [13, 14]. Here, we describe a potentially preventative and therapeutic compound with binding consistent with avidity to all currently identified SARS-CoV-2 VOCs, reduction of virus-induced lung inflammation, and penetration of enzymatically active drug to peripheral tissues. As G921 incorporates the binding domain of the natural receptor for pathogenic SARS-CoV-2, current and future SARS-CoV-2 VOCs are unlikely to develop resistance to G921 unless the virus evolves to use a different primary receptor other than ACE2.

## METHODS

### Analysis of Binding to SARS-CoV-2 Spike Proteins and Virus Variant Spike Proteins

Binding analysis was performed using a ForteBio Octet Red biolayer interferometry system (BLI, Sartorius, France). G921 concentrations used were 25, 12.5, and 6.25, 3.13, 1.56, and 0.78 µg/mL. Commercial recombinant His-tagged viral variants were loaded onto anti-His sensors from ForteBio (HIS1K; cat# 18-5121) in 1X kinetics buffer for 300 seconds and transferred to buffer for baseline measurement (60 seconds). On rate was measured for 300 seconds after transfer of sensor tip to G921 in kinetics buffer. Off rate was measured for 600 seconds by transfer of sensor tip to kinetics buffer. The binding parameters were calculated by ForteBio Data Analysis 6.4 software module using measured on and off rates and a 1:1 model fit. Spike proteins were from ACRO-Biosystems and were derived from the SARS-CoV-2 spike protein parental sequence (GenBank: QHD43416.1). Each of the derived sequences contain proline substitutions (F817P, A892P, A899P, A942P, K986P, V987P) and alanine substitutions (R683A and R685A) introduced to stabilize the trimeric prefusion state of SARS-CoV-2 S protein and abolish the furin cleavage site, respectively. The proteins tested were SARS-CoV-2 wildtype (Wuhan-Hu-1; cat# SPN-C52H9), B.1.1.7 Alpha (cat# SPN-C52H6), B.1.351 Beta (cat# SPN-C52Hk), P.1 Gamma (cat# SPN-C52Hg), B.1.617.2 Delta (cat# SPN-C52He), and B.1.617.1 Kappa (cat# SPN-C52Hr).

**Focus Reduction Neutralization Assay.** The direct neutralization of SARS-CoV-2 by G921 was tested in Vero E6 cells. Virus stock of SARS-CoV-2 USA-WA1/2020 isolate, (NR-52281, BEI) was performed by infecting Vero E6 cells (ATCC, CRL, 1586) at an MOI of 0.1 in MEM medium (Corning, CellGro) supplemented with 2% fetal bovine serum. Virus-containing medium was collected at day 3 post infection after appearance of cytopathic effects. The titers of viral seed stocks (passage #2) were measured by TCID_50_ using a focus-forming assay. In addition, an aliquot of virus stock was sequenced, and no major mutation/deletion was observed.

Vero E6 cells were seeded in a 96-well plate (15,000 cells per well) and cultured at 37°C, 5% CO_2_ for 16-18 hours. Next day, serially diluted G921 was incubated with previously titrated (MOI of 0.01 or ~50-70 foci/well) SARS-CoV-2 USA-WA1/2020 isolate (NR-52281, BEI) for 1 hour at 37°C. This mixture was used to infect grown Vero E6 cells for 1 hour, followed by the addition of overlay media (Opti-MEM, 2% FBS, 2.5 μg/mL amphotericin B, 20 μg/mL Ciprofloxacin, 2% methylcellulose) and then incubated for 3 days for the foci assay. Controls include uninfected cells, infected cells, and infected cells incubated with a primate convalescent serum to SARS-CoV-2 collected at 21 days post infection. After 3 days incubation, the amount of antigen present in cells was measured by using a monoclonal anti-SARS coronavirus recombinant human IgG1 labeled with HRP, clone CR3022 (BEI NR-52392), and foci were visualized and imaged using True Blue HRP substrate and ELISpot reader (CTL) with manual counting. In addition, cytotoxicity of G921 was measured in Vero E6 cells using the CellTiter 96® Non-Radioactive Cell Proliferation (Promega) kit. The experiments with live virus were performed in a biosafety Level 3 (BSL3) laboratory according to “Biosafety in Microbiological and Biomedical Laboratories” (HHS Publication CDC938395), Emory Standard Operating Procedures, and safety manuals.

### Efficacy Testing in Golden Syrian Hamsters

Male golden Syrian hamsters were purchased from Charles River Laboratories at 5-6 weeks of age and housed in sterile micro-isolator cages with sterile food and water ad libitum. Hamsters were maintained according to the guidelines of the Animal Welfare Act. All animal care procedures were performed according to protocols approved by the UTHSC animal care and use committee. Hamsters were implanted with microchips, weighed, and randomly assigned to 1 of 5 groups (n=4). The hamsters were intranasally infected with SARS-CoV-2 (2019-nCoV/USA-WA1/2020) at 2.0 × 10^4^ plaque forming units (PFUs) at day 0. The hamsters were treated with G921 at 10, 30, and 70 mg/kg or phosphate buffered saline (PBS) as control by subcutaneous injection on day 1, 2 hours prior to infection on day 0, and day 2. Clinical signs, temperature, and weight of animals were measured daily. At day 7 post infection, hamsters were anesthetized by ketamine (250 mg/kg) + xylazine (100 mg/kg) via intraperitoneal injection and then euthanized by intraperitoneal Euthasol, 200 µL/100 g, and the lungs collected for histopathology. SARS-CoV-2 was from BEI Resources (ATCC No. NR-52281) and seed stocks were grown in Vero E6 TMPRSS cells and sequenced. All *in vivo* experiments were performed in Animal Biosafety Level 3 conditions within the UTHSC Regional Biocontainment Laboratory according to UTHSC RBL Standard Operating Procedures and safety manuals.

## PATHOLOGY METHODS AND SCORING

Hematoxylin and Eosin staining was performed, and slides were digitized at UTHSC (VS200 Slide scanner, Olympus). Histological assessment was performed by a board-certified veterinary pathologist using Aperio ImageScope software. The inflamed fraction of diseased lung in affected animals was scored manually by outlining the total lung area, followed by diseased area. Diseased area was defined as nodular areas of epithelial proliferation and inflammation, which efface normal lung parenchyma. The fraction of diseased lung was then calculated by dividing diseased area by total lung area for each animal. Histological assessment of vascular damage in diseased lung in affected animals was performed by assessing 6 parameters of vascular injury (perivascular inflammation, perivascular edema, intramural inflammation, intramural necrosis, intramural fibrin deposition, and tunica media vacuolation). Each parameter was scored 0–2 for each Syrian hamster. Parameters indicative of more mild vascular damage were given a score of 1 if present, 0 if absent. Parameters indicative of more severe vascular damage were given a score of 2 if present, 0 if absent. The histopathologist was unaware of the treatment group.

### Rat Pharmacokinetics, Bronchoalveolar Lavage Fluid Analysis, and Urine Analysis

For pharmacokinetic studies in rat Wistar strain rats (ENVIGO, female, 200-220 g), 3 animals per group in each of 4 groups were dosed either intravenously or subcutaneously with 20 or 60 mg/kg and serum collected at 0.25, 4, 8, 24, 72, 120, 168, and 240 hours. G921 levels were measured using an electrochemiluminescent multiplexed immunoassay (ECL-MI, Meso Scale Discovery, MD) with capture antibody polyclonal rabbit anti-human IgG4 (MyBioSource cat# MBS2028130) and detecting antibody rabbit anti-human IgG Fc, biotin conjugate (Invitrogen cat# 31789) followed by Streptavidin SULFO-TAG (MSD cat# R32A-5). In a separate study, rats (n = 4/group) were injected intravenously with 100 mg/kg G921 or PBS for control and placed in metabolic cages for urine collection. Twenty-four hours later, the urine volume was recorded. After exsanguination, the trachea was exposed, cannulated, and the lungs washed twice with 5 mL heparinized PBS for bronchoalveolar lavage fluid (BALF) collection. G921 levels were measured in the BALF and urine of rats by ECL-MI. ACE2 enzymatic activity was measured using a Fluorometric Angiotensin II Converting Enzyme Activity Assay Kit using the manufacturer's protocol (Biovision, CA).

## RESULTS

### Protein Expression and Analysis

G921 was assessed by non-reduced and reduced SDS-PAGE (Supplementary Figure 1A) and size exclusion chromatography (SEC) (Supplementary Figure 1B) after purification. Non-reduced SDS-PAGE demonstrates an upper band below 260 kD that corresponds to the dimeric form of G921 and a lower band at approximately 120 kD that likely represents the monomeric form of G921. Reduced SDS-PAGE demonstrates a band at approximately 120 kD that corresponds to the monomeric form of G921. SEC-HPLC of G921 reveals 1 major peak representing the dimeric form of G921. The right shoulder of the major peak likely represents the monomeric form of G921.

### G921 Binding to SARS-CoV-2 Spike Protein from Viral Variants

**Figure 1.**
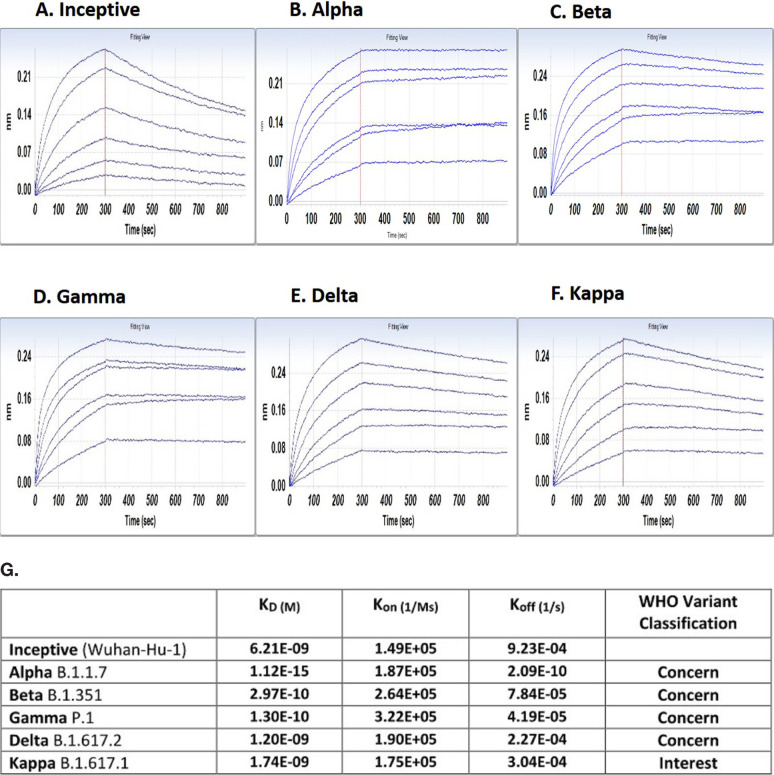
**Binding of G921 to viral spike protein variants by bio-layer interferometry**. (A) Binding curve for inceptive isolate (Wuhan-Hu-1). (B-F) Variants of concern or variant of interest. (G) G921 binding parameters of the spike protein of SARS-CoV-2 variants relative to the inceptive (Wuhan-Hu-1) isolate as determined by bio-layer interferometry using a 1:1 binding model. Classification of viral variants in right column.

**Figure 2.**
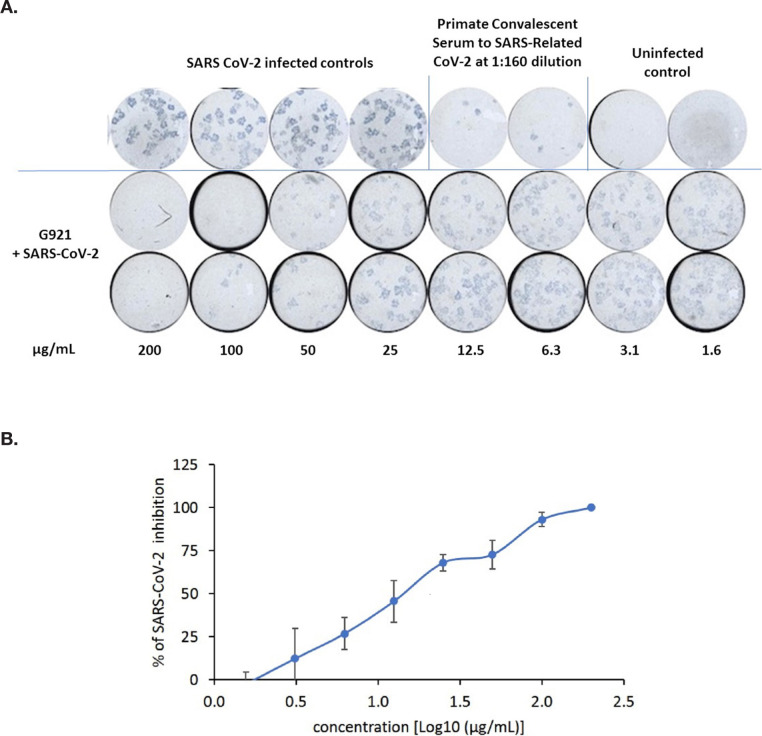
(A) Focus reduction neutralization assay using SARS-CoV-2 Wuhan-Hu-1 assessing a range of concentrations of G921 from 1.6 to 200 µg/mL with positive and negative controls. (B) Plot of G921 percent inhibition of SARS-CoV-2 foci in a dose-dependent manner.

BLI demonstrates G921 binding to the inceptive SARS-CoV-2 Wuhan-Hu-1 spike protein with a dissociation coefficient of 9.23E-04 (Figure1A). SARS-CoV-2 variants with increased infectivity of concern to the WHO have been isolated. Binding of G921 to the spike protein of Alpha, Beta, Delta, Kappa, and Gamma variants is shown in Figure 2 B-F. Each WHO viral VOC demonstrates increased binding to G921 relative to the inceptive isolate, as indicated by the lowered K_D_ values and dissociation rates (Figure 1G). The data for the Omicron variant are similar showing increased binding relative to inceptive (not shown). The lower K_D_ values and lower dissociation rates are consistent with reports that the viral spike protein has evolved to bind more strongly to the human ACE2 receptor [13, 14], and that same increased binding can also be observed for the G921 ACE2 Fc fusion protein.

### Neutralization Activity of G921 Against SARS-CoV-2

A focus reduction neutralization assay was used to measure the ability of G921 to neutralize SARS-CoV-2 *in vitro*. As expected, the positive control convalescent serum to SARS-CoV-2 exhibits neutralizing antibodies with cell protection from SARS-CoV-2 infection at 1:160 dilution (Figure 2A). A representative photograph of the focus-reducing assay demonstrates that the entry of SARS-CoV-2 virions in the cells likewise was inhibited by G921 in a concentration-dependent manner. The 50% and 90% effective concentration (EC_50/90_) of G921 required to inhibit viral protein expression was calculated by nonlinear regression analysis (Figure 2B) with EC_50/90_ values of 16.3±4.2 and 94.3±13.3 µg/mL, respectively, and a complete inhibition of infection of cells at 200 µg/mL. In addition, G921 does not exhibit cytotoxicity in Vero E6 cells when tested at concentrations up to 200 µg/mL in a 4-day MTS cell proliferation assay. The experiment was done twice, and representative results shown.

### Effects on Golden Syrian Hamsters

**Figure 3.**
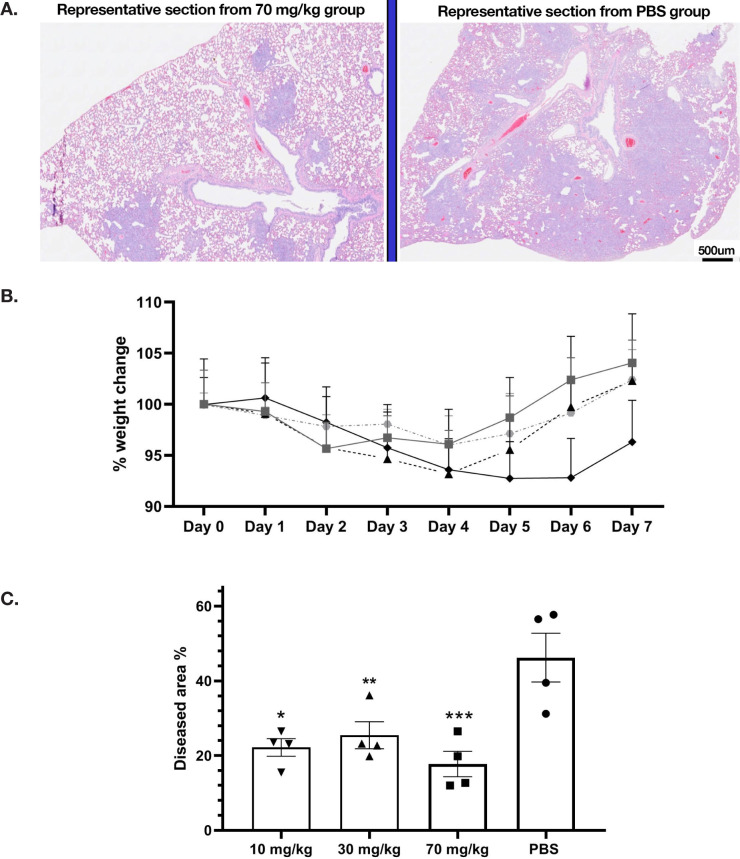
**Golden Syrian Hamster Efficacy study**. (A) Representative histopathology slides from 70 mg/kg and PBS groups after unblinding, slide scale bar is 500 µm. (B) Percent weight changes of treated compared to mock-treated golden Syrian hamster G921 efficacy study. Legend: 70 mg/kg group = rectangles, 30 mg/kg = triangles, 10 mg/kg = circles, virus only = diamonds. Four animals were treated per group (mean +SEM). (C) Quantitation of histopathology data (mean +/- SEM), magnification assessed 1X-40X. The 10, 30, and 70 mg/kg groups demonstrate reduction of total lung inflammation relative to PBS. (Student t-test *P* values 10 mg/kg * 0.0132, 30 mg/kg ** 0.0317, and 70 mg/kg *** 0.0082).

The golden Syrian hamster model of SARS-CoV-2 infection was used to measure the efficacy of G921. The hamsters were weighed and monitored daily. Histology was performed on animals at day 7 post-infection. Figure 3A demonstrates representative histopathology slides from 70 mg/kg and PBS groups. Hamsters infected with SARS-CoV-2 but not treated with G921 lost weight beginning day 2 post-infection, exhibited maximal weight loss on day 5 post-infection (-7.45±9.4 g), and began to regain weight after day 6 post-infection (Figure 3B). Hamsters treated with 70 mg/kg of G921 exhibited less weight loss (-4.25±1.5 g) compared to the virus-only group. This group demonstrated maximal weight loss on day 2 post-infection, began to regain weight on day 3 post-infection, and had regained weight to near baseline by 5 days post-infection. Weight differences between treatment groups did not reach statistical significance due to significant inter-animal variation and small group size. Areas of lung inflammation were quantitated (Figure 3C). Lungs from G921-treated groups are associated with quantitative reductions in lung inflammation and proliferation relative to the virus-only group with statistical significance in all groups relative to PBS.

**Figure 4.**
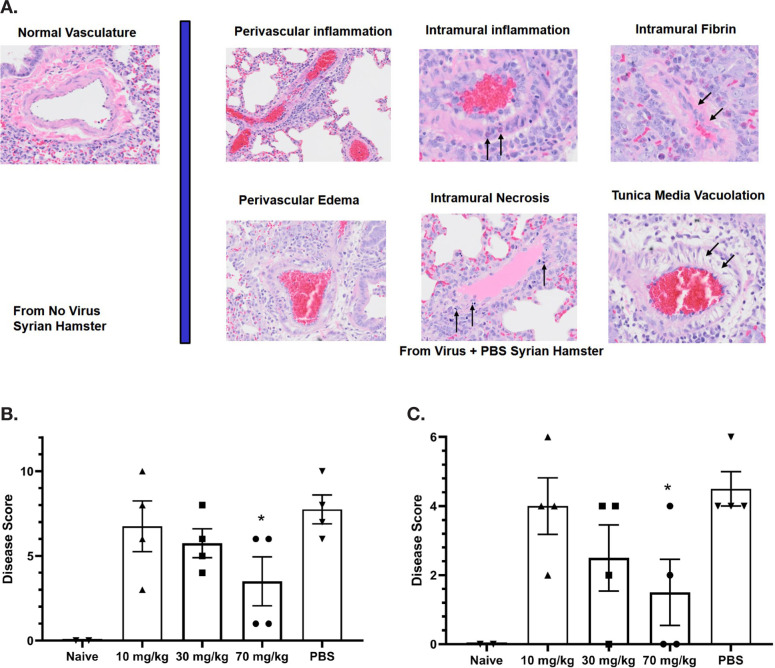
**Assessment of Lung Pathology in the Syrian Hamster Efficacy Study**. (A) Representative vascular histopathology slides from control (no virus) and virus plus PBS groups. Arrows indicate inflammatory cells within the blood vessel wall (intramural inflammation), necrotic cellular debris within the vessel wall (intramural necrosis), hypereosinophilic proteinaceous deposits within the vessel wall (intramural fibrin), and vacuolation of smooth muscle cells of the tunica media. (B) Quantitation of lung vascular histopathology data (mean +/- SEM). Quantitation used magnification 2X-40X. The 70 mg/kg group is associated with reduction of lung vascular damage relative to PBS (*student t-test *P* value = 0.044). (C) Quantitation of lung intramural vascular histopathology data (intramural inflammation, necrosis, fibrin) (mean +/- SEM). The 70 mg/kg group is associated with reduction of lung intramural vascular damage relative to PBS (* student t-test *P* value = 0.032).

A detailed analysis of differential effects on vasculature was performed by semi-quantitative scoring of histopathological markers of vascular injury (Figure 4A), including perivascular inflammation, intramural inflammation, intramural fibrin deposition, perivascular edema, intramural necrosis, and tunica media vacuolation. With 4 animals per cohort, the decrease in lung vascular damage and vasculitis at 7 days post-infection trended dose-responsive with statistical significance (*P* = 0.044) for the 70 mg/kg group relative to the PBS-treated group, indicating significant differences between treated and untreated hamsters (Figure 4B). Three lung vascular intramural pathology parameters were analyzed for each Syrian hamster (Figure 4C). With 4 animals per cohort, the decrease in lung vascular intramural damage and vasculitis at 7 days post-infection trended dose-responsive with statistical significance (*P* = 0.032) for the 70 mg/kg group relative to the PBS-treated group, indicating significant differences between treated and untreated hamsters.

### Efficacy Testing in H1N1 Mouse Influenza Model

**Figure 5.**
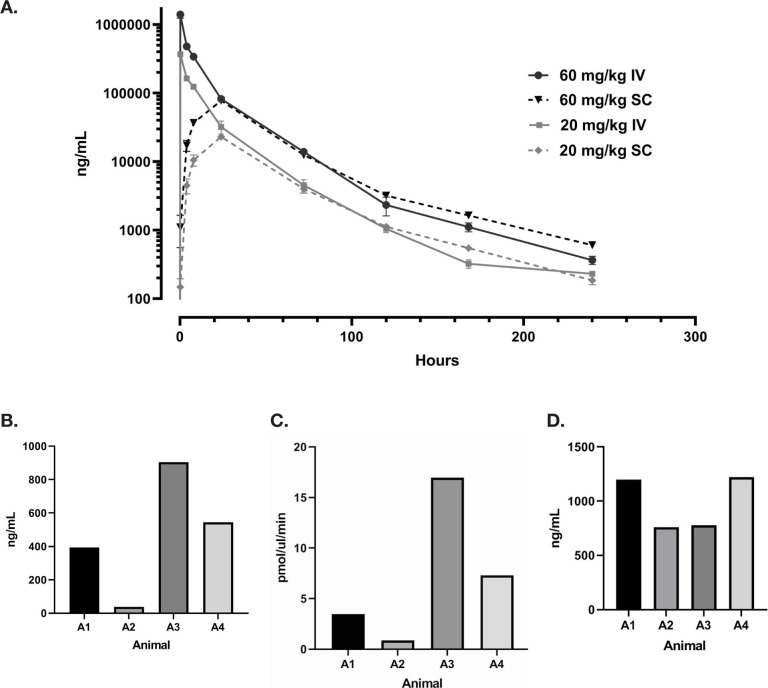
**Rat pharmacokinetics study**. (A) G921 levels assessed by ECL-MI in serum samples after intravenous and subcutaneous dosing at 20 mg/kg and 60 mg/kg each. Serum levels of G921 (mean +/- SEM). Rat study assessing exposure of G921 in lung and urine. (B) G921 levels (ng/mL) measured in rat BAL fluid by ECL-MI. Animal-to-animal variation possibly due to variation in BAL extraction technique. (C) ACE2 enzyme activity levels in rat BAL fluid measured by ACE2 enzyme activity assay (pmol/μL/min). (D) G921 levels (ng/mL) measured by ECL-MI in rat urine. A1-A4 represents individual animals.

Efficacy testing of G921 in a mouse influenza model was performed at Melior Discovery (Exton, PA). Virus-infected drug-treated and PBS control groups were established for day 7 histology testing (9 per group) and for lethality assessment by day 14 (11 per group). Inoculation of animals with A/PR/8/34 H1N1 influenza virus (6.1 Lg EID_50_ in 50 μL) produced a severe disease. There were 0% lethal effects by day 7 after infection in all groups. There was 91% lethality at day 14 after infection in the PBS-treated group and 82% in the G921-treated group. Animals treated with vehicle and a 50 μL dose of A/PR/8/34 H1N1 had a median survival of 11 days, while animals dosed with A/PR/8/34 H1N1 and treated with G921 had a median survival of 12 days (not statistically significant). Histology analysis of lungs from the day 7 cohorts demonstrated a statistically significant decrease in total inflamed lung area in G921-treated animals relative to vehicle-only treated animals. (Supplementary Methods and Figure 5A–D).

### G921 Rat Pharmacokinetics and G921 Levels and Enzymatic Activity in BAL Fluid

Rats were dosed intravenously and subcutaneously with 20 or 60 mg/kg of G921 and serum G921 levels were measured at indicated time points (Figure 5A). The terminal half-life of G921 is approximately 28 hours in rats. No significant differences in serum G921 levels are observed between intravenous and subcutaneous administration after 24 hours. In a second study, rats administered 100 mg/kg of G921 or PBS control intravenously had G921 levels measured in urine and BALF and quantitation of ACE2 enzymatic activity. All rats were individually examined, and no injury, disease, or clinical distress was identified including behavior or feeding activity. G921 was recovered from the BALF of rats (Figure 5B) and exhibited proportional enzymatic activity (Figure 5C), indicating that G921 successfully traverses intact from blood into lung. The animal-to-animal difference observed is likely due to differences in post-mortem BALF fluid recovery. G921 was also detected in the urine by ECL-MI (Figure 5D), indicating that G921 is excreted by the kidneys.

### G921 Cynomolgus Monkey Pharmacokinetics

The pharmacokinetic profile of G921 was determined following a single intravenous infusion or subcutaneous administration to the cynomolgus monkeys at doses of 10 mg/kg, 30 mg/kg, or 100 mg/kg intravenously or 100 mg/kg subcutaneously, 1M + 1F per group. As shown in Supplementary Figure 2, after 24 hours there is little difference in serum levels of G921 between the intravenous and subcutaneous administration routes, suggesting that subcutaneous dosing may be feasible. G921 terminal half-life is estimated to be 44.1 hours and 41.0 hours in the male and female macaque, respectively, when administered by subcutaneous injection, and 44.7 hours to 79.7 hours in males and 47.6 hours to 73.4 hours in females when administered via intravenous infusion. Over the dose range, exposure to G921 increases dose-dependently. No adverse effects were observed in the study.

### G921 Binding to FcγRs and FcRn

Antibody dependent enhancement (ADE) has been demonstrated to be implicated in the pathogenesis of certain coronaviruses [15]. Data from the study of SARS-CoV-1 and other respiratory viruses suggest that anti-SARS-CoV-2 antibodies could exacerbate COVID-19 through ADE [16], although this concept is contested by others. ADE occurs when antibodies recognize the virus and simultaneously bind to host cell FcγR through the Fc domain. Although Fc-mediated infectivity of SARS-CoV-2 is a theoretic and not yet demonstrated possibility, G921 was designed with intent to reduce the possibility of an ADE effect through the Fc domain by utilizing IgG4 Fc in the fusion protein. Binding to high affinity Fc gamma receptor I and the low affinity Fc gamma receptors by BLI are shown in Supplementary Figure 3. No binding of G921 to FcγRIIa, FcγRIIb, and FcγRIIIa is observed. However, binding is observed for the recombinant IgG1 Fc control G001. Binding of G921 to the high-affinity receptor FcγRI is retained. In contrast to the lack of binding to the low-affinity receptors, G921 also retains binding to the neonatal receptor FcRn. G921 binds equally well to the FcRn compared to recombinant IgG1 Fc control, potentially facilitating transcytosis of G921 from circulation to peripheral tissue and extracellular space through Fc domain interaction with FcRn.

### G921 ACE2 Activity

ACE2 has potent anti-inflammatory activity as outlined above. We sought to determine whether the ACE2 ECD fragments in G921 retained ACE2 enzymatic activity. G921 demonstrates ACE2 enzymatic activity at a level similar to a positive ACE2 control that was expressed in HEK cells (Supplementary Figure 4).

## DISCUSSION

The Fc fusion protein G921 with 2 ACE ECD fragments binds well to all current WHO designated VOCs, as expected, since pathogenic variants of SARS-CoV-2 to date bind increasingly well to the cell surface enzyme and viral receptor ACE2 [13, 14]. As the natural receptor for pathogenic SARS-CoV-2 is ACE2 [1–5], the virus is unlikely to be able to evolve to have decreased binding to ACE2 without simultaneously losing pathogenicity, unless the virus evolves to bind to a different receptor. G921 also incorporates 2 copies of the receptor binding domain of ACE2 leading to binding of spike protein consistent with avid binding. Future SARS-CoV-2 VOCs are equally unlikely as current variants to develop decreased binding to ACE2 or resistance to G921.

G921 builds on prior work by others who have contemplated ACE2 as a therapeutic, both as an isolated protein and as an Fc fusion protein, which has been reviewed well by Kruse [17]. What we believe may be unique to G921 is combining an ACE2 ECD fragment with an IgG4 Fc, in both cases to enhance transit to lung and other organs, while avoiding the potential of ADE.

We show that G921 neutralizes viral infection of Vero E6 cells in a concentration-dependent manner without cytotoxicity and with an EC_50_ of 16 µg/mL. The lowest dose associated with observed efficacy *in vivo* in the Syrian Hamster model is 10 mg/kg, which, assuming 14 mL blood per animal, indicates pharmacokinetic parameters in a similar range to the estimated EC_50_ of the G921 *in vitro* neutralization assay. This contrasts with the several log order disparity of potency for anti-SARS-CoV-2 monoclonal antibodies between the *in vitro* neutralization assay and dosing levels associated with observed efficacy *in vivo* [18–20].

SARS-CoV-2 infection diminishes cell surface ACE2, believed to be a critical modulator of inflammatory pathways, while G921 is designed not only to bind to all SARS-CoV-2 VOCs but also to access peripheral tissue and replenish diminished ACE2 enzyme activity. Deficiency of cell-membrane ACE2, an enzyme not normally consumed in the hydrolysis process but consumed in the process of SARS-CoV-2 cell entry, diminishes conversion of Ang II to Ang-(1-7). A decrease in ACE2 with a corresponding increase in the ratio of Ang II to Ang-(1-7) will upregulate the inflammatory AT1 receptor pathway while simultaneously downregulating the Ang-(1-7)-driven Mas-receptor anti-inflammatory pathway, a dual hit. A parallel process occurs with the inability to hydrolyze des-Arg^9^-bradykinin [21], which binds the inflammatory kinin B1 receptor. The known inflammatory outcomes of ACE2 deficiency are consistent with the observed inflammatory pathology associated with COVID-19 though causation has still not been established.

Influenza virus H1N1 downregulates the expression of ACE2 in mice [22] and recombinant ACE2 ameliorates influenza H5N1 virus-induced lung injury [23]. As the H1N1 influenza model is ACE2-receptor independent, the observed reduction in lung inflammation in the H1N1 influenza model following G921 treatment (Supplemental Figure 5) indicates an effect of G921-supplied ACE2 enzymatic activity and supports that G921 can work as a direct anti-inflammatory molecule in diseases independent of the ACE2-driven direct binding and neutralization.

Whether ADE is a factor in SARS-CoV-2 infections remains a controversial topic with advocates arguing for [24] and against [25] the possibility. We elected to engineer G921 to benefit from the transcytosis and extended half-life potential of IgG4 Fc while eliminating binding to low-affinity FcγRs and thereby avoiding any likelihood of ADE of G921 that could theoretically occur with an IgG1 Fc fusion protein.

In contrast to the diminished binding to FcγRs, G921 retains binding to the neonatal receptor. FcRn functions as a recycling or transcytosis receptor, bidirectionally transporting ligands across polarized cellular barriers [26]. The findings that G921 is present in BALF and urine and that ACE2 enzyme activity also was present to a corresponding degree indicate that the FcRn binding may facilitate transport to extravascular space, which could be important not only in neutralizing SARS-CoV-2, but also in restoring potentially deficient peripheral ACE2 activity. Truncation of ACE2 ECD has been employed to increase penetration of enzymatically active ACE2 (without Fc fusion) into urine [27]. When infused to mice with genetic ACE2 deficiency, a single intravenous injection of ACE2 1-619 resulted in detectable ACE2 activity in urine, whereas infusion of the native ACE2 did not. G921 demonstrates similar findings in the context of a homodimeric Fc fusion protein.

G921 is engineered for delivery to peripheral tissues and is recovered enzymatically active from peripheral tissues. These functional attributes likely account at least in part for the anti-inflammatory activity of G921 in protecting Syrian hamster weight and Syrian hamster lungs from virus-induced lung damage. The protective effect for SARS-CoV-2-induced weight loss is of similar magnitude to that reported by monoclonal antibodies [16] but with a later onset of weight gain.

G921 has several other attributes as a potential COVID-19 therapeutic. Pharmacokinetic studies in the cynomolgus monkeys as well as in rats demonstrate a sufficiently long serum half-life to allow for longer dosing intervals and possible subcutaneous dosing. An initial Syrian hamster therapeutic *in vivo* study with G921 indicated a 1-log reduction in viral load in the hamster nasal passages at day 4 with high inter-individual variability, likely related to the small number of animals tested (data not shown). The reduced lung and endovascular inflammation observed in the Syrian hamster prophylactic *in vivo* model could be attributed to a direct-acting antiviral effect through binding and neutralization of the viral spike protein or to an anti-inflammatory ACE2 replacement therapy mediated through a decrease in the Ang II to Ang-(1-7) ratio and subsequent beneficial effects on the ATR1 and Mas-receptor pathways. Determination of the precise contribution of these effects in reducing inflammation requires further investigation.

Recombinant ACE2 has been previously in development as a therapeutic for vascular diseases [28] and more recently has been in clinical trial as a treatment for COVID-19 [29], and, in all cases, the drug was well tolerated without drug-related adverse events [30, 31]. G921 binds well to SARS-CoV-2 VOCs, is delivered to peripheral tissues as a result of both its truncated ACE2 ECD and FcRn-mediated transcytosis, has a much longer half-life than recombinant ACE2 as a result of being an Fc fusion protein, is associated with significant reduction in inflammatory response to SARS-CoV-2 *in vivo*, and is unlikely to be associated with ADE. G921 was cloned and expressed in a high yield stable cell CHO expression system with titers obtained after protein A affinity purification approaching 7 g/L.

Numerous other disease conditions are associated with increases in Ang II indicating insufficient ACE2, notably influenza-associated acute lung injury. G921 may also be assessed in other diseases characterized by absolute or relative ACE2 deficiency.

## SUPPLEMENTARY DATA

Supplementary materials are available at the *Pathogens and Immunity* website. Supplementary data may be provided by the authors to benefit the reader. Supplementary data are not copyedited and are the sole responsibility of the authors. Questions or comments related to supplementary materials should be addressed to the corresponding author.
