# Supplementary material for: An ACE2-IgG4 Fc Fusion Protein Demonstrates Strong Binding to All Tested SARS-CoV-2 Variants and Reduced Lung Inflammation in Animal Models of SARS-CoV-2 and Influenza
Source: Pathog Immun. 2022 Aug 23;7(1):104–21. doi: 10.20411/pai.v7i1.491 (PMC9438944; doi:10.20411/pai.v7i1.491)
Supplement: Supplemental Methods and Figures [file pai-7-104-s01.pdf]

PATHOGENS AND IMMUNITY
IN ASSOCIATION WITH CASE WESTERN RESERVE UNIVERSITY AND THE RICHARD J. FASENMYER FOUNDATION

SUPPLEMENTARY FIGURES AND TABLES

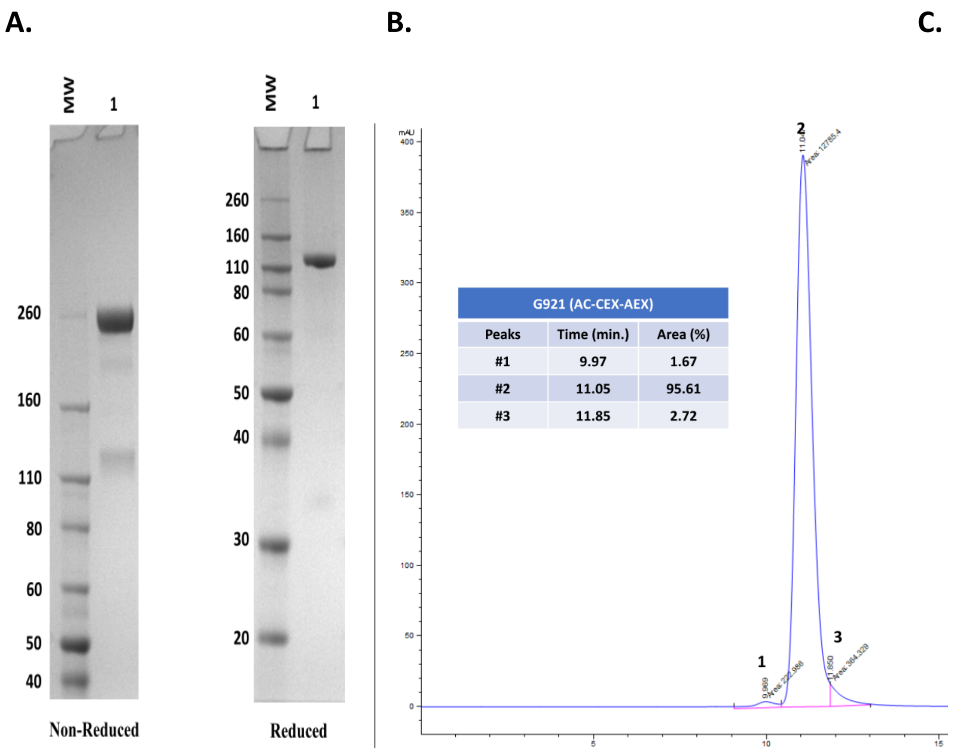

G921 (AC-CEX-AEX)		
Peaks	Time (min.)	Area (%)
#1	9.97	1.67
#2	11.05	95.61
#3	11.85	2.72

Figure 1. Characterization of G921 purified protein by (A) non-reduced and reduced SDS-PAGE (B) SEC-HPLC and (C) schematic diagram of G921 showing the IgG4 Fc fused to two ACE2 extracellular domains.

Merigeon EY, Yang D, Ihms EA, Bassit LC, Fitzpatrick EA, Jonsson CB, Schinazi RF, Block DS, Olsen HS. An ACE2-IgG4 Fc Fusion Protein Demonstrates Strong Binding to All Tested SARS-CoV-2 Variants and Reduced Lung Inflammation in Animal Models of Both SARS-CoV-2 and Influenza. *Pathogens and Immunity*. 2022;7(1): 104-121. doi: 10.20411/pai.v7i1.491

PATHOGENS AND IMMUNITY
IN ASSOCIATION WITH CASE WESTERN RESERVE UNIVERSITY AND THE RICHARD J. FASENMYER FOUNDATION

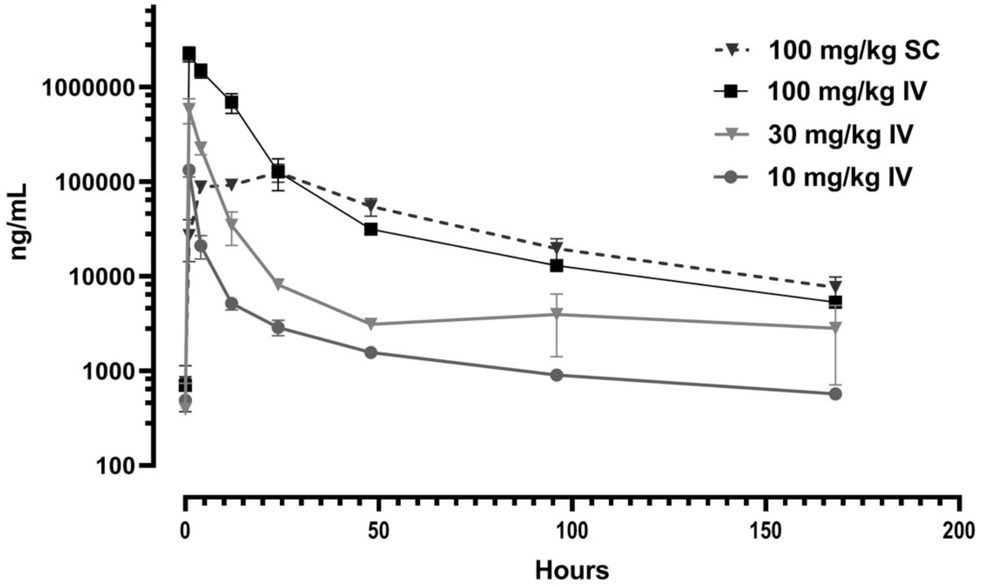

Figure 2. Cynomolgus pK study. G921 levels assessed in serum samples by ECL-MI after intravenous (IV) dosing at 10, 30, and 100 mg/kg and after subcutaneous (SC) dosing at 100 mg/kg. Serum levels of G921 (mean +/- SEM).

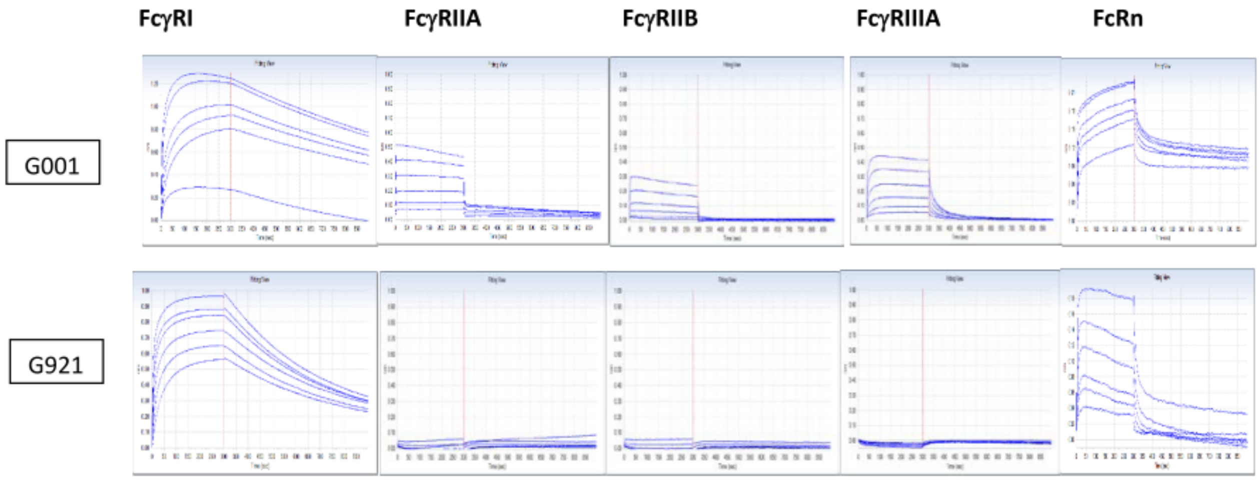

Figure 3. G921 binding to Fcγ receptors by BLI. Binding curves for G921 binding to Fcγ receptors and FcRn compared to recombinant IgG1 Fc (G001) control.

PATHOGENS AND IMMUNITY
IN ASSOCIATION WITH CASE WESTERN RESERVE UNIVERSITY AND THE RICHARD J. FASENMYER FOUNDATION

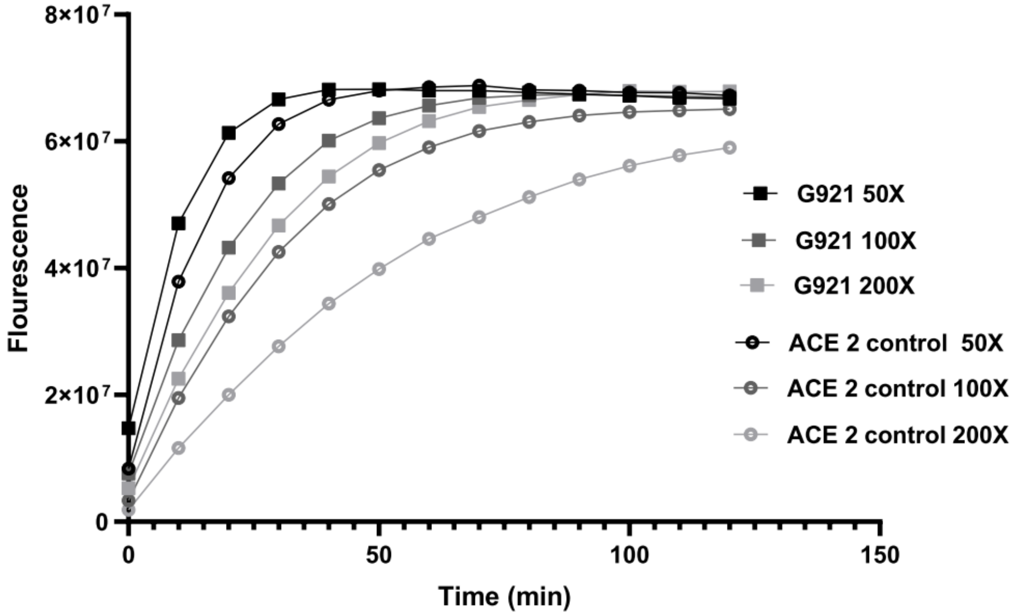

Figure 4. ACE2 enzymatic activity as assessed by the Biovision ACE2 enzyme activity assay kit. G921 demonstrates ACE2 enzymatic activity at a level similar to a positive ACE2 control.

Merigeon EY, Yang D, Ihms EA, Bassit LC, Fitzpatrick EA, Jonsson CB, Schinazi RF, Block DS, Olsen HS. An ACE2-IgG4 Fc Fusion Protein Demonstrates Strong Binding to All Tested SARS-CoV-2 Variants and Reduced Lung Inflammation in Animal Models of Both SARS-CoV-2 and Influenza. *Pathogens and Immunity*. 2022;7(1): 104-121. doi: 10.20411/pai.v7i1.491

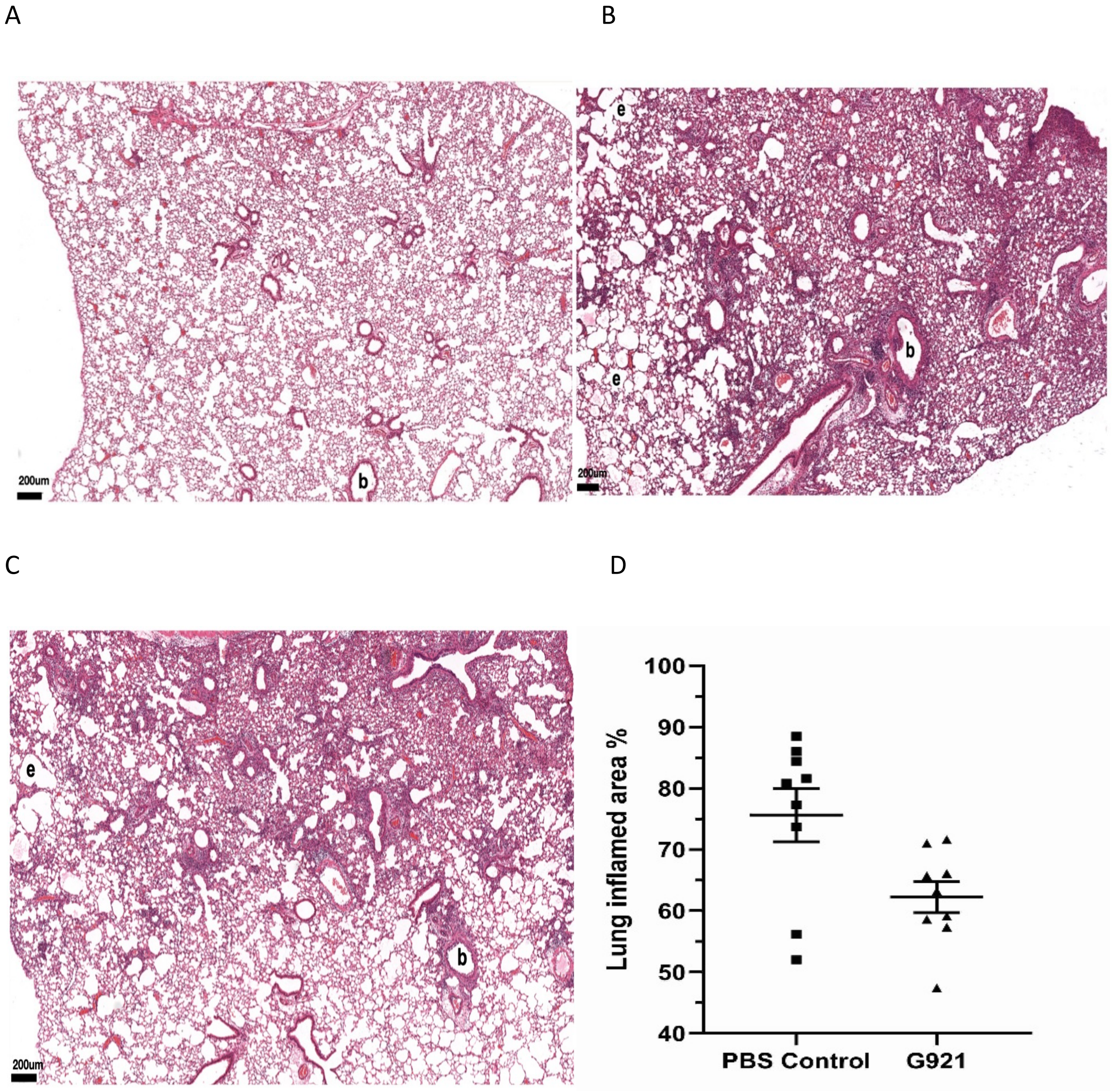

Figure 5. G921 efficacy study in H1N1 Influenza model. Histological analysis of lungs performed at day 7 following infection. (A) Uninfected PBS control (normal lung). (B) Representative lung from infected animal treated with vehicle (PBS) control. There is severe bronchointerstitial pneumonia, with prominent peribronchiolar (b-bronchiole) involvement and thickening of the alveolar septae. There are frequent areas of emphysema (e) adjacent to areas of severe inflammation, suggestive of severe clinical disease and respiratory distress. Areas of normal lung tissue are scarce. (C) Representative lung from infected animal treated with G921.

Merigeon EY, Yang D, Ihms EA, Bassit LC, Fitzpatrick EA, Jonsson CB, Schinazi RF, Block DS, Olsen HS. An ACE2-IgG4 Fc Fusion Protein Demonstrates Strong Binding to All Tested SARS-CoV-2 Variants and Reduced Lung Inflammation in Animal Models of Both SARS-CoV-2 and Influenza. *Pathogens and Immunity*. 2022;7(1): 104-121. doi: 10.20411/pai.v7i1.491

PATHOGENS AND IMMUNITY
In Association with Case Western Reserve University and The Richard J. Fasenmyer Foundation

There is moderate bronchointerstitial pneumonia similar in histology to infected + PBS control animals but less extensive, with interstitial and peribronchiolar involvement. Areas of inflammation are smaller, and emphysema is present but is less frequent, and less severe as in this image. Areas of normal lung tissue can often be seen between areas of inflammation which differs from PBS control. (D) Quantitation of histology data (mean +/- SEM). G921 treated group demonstrates reduced lung inflammation area relative to infected animal treated with PBS (*P*=0.0169 student t test).

SUPPLEMENTARY METHODS

G921 Cloning and Protein Expression

Codon optimized DNA sequence encoding G921 containing amino acid 19-615 of the ACE2 extracellular domain with a leader peptide from murine light chain and directly fused to the IgG4 Fc at the hinge domain was cloned into expression vector pXLG6. A stable CHO cell pool expressing G921 was established from which a stable cell line clone was isolated and employed for G921 expression (ExcellGene SA, Monthey, Switzerland). G921 was purified from stable cell line supernatant using a combination of protein A affinity and ion exchange chromatography. Non-reduced and reduced SDS-PAGE analysis of G921 was performed using NuPAGE 3-8% Tris-Acetate and 4-12% Bis-Tris SDS gels respectively. Analytical size exclusion chromatography was done on an Agilent Bio SEC-3, 300Å column in 50 mM MES, 0.2M Arginine pH 6.0. Dynamic light scattering studies using a Stunner instrument (Unchained Labs, Pleasanton, CA) confirmed that G921 has a molecular weight consistent with a homodimeric molecule.

Cynomolgus Monkey pk study

Non-compartmental analysis of G921 concentrations in serum was performed by using the Phoenix WinNonlin 6.4 software. Maximum G921 serum concentration was reached 24 hours post subcutaneous dose or immediately post end of IV infusion. G921 serum concentrations declined at an estimated $t_{1/2}$ of 44.1 and 41.0 hours in the male and female, respectively when administered by subcutaneous injection, and at an estimated $t_{1/2}$ of 44.7 to 79.7 hours in males and 47.6 to 73.4 hours in females when administered via IV infusion. Over the dose range, exposure to G921 increased dose-dependently and in a more-than dose proportional manner in both sexes and both routes of administration. There were no evident sex-related differences in any of the measured pharmacokinetic parameters. When comparing the subcutaneous versus intravenous infusion routes of administration at 100 mg/kg, systemic exposure from IV infusion route animals were 3.3 to 18.8-fold higher than from subcutaneous route animals

PATHOGENS AND IMMUNITY
In Association with Case Western Reserve University and The Richard J. Fasenmyer Foundation

ACE2 Enzymatic Activity

In vitro ACE2 enzymatic activity was assessed using the Biovision ACE2 Activity Assay Kit (cat #K897-100). ACE2 cleaves a synthetic MCA fluorophore-based peptide substrate to release a free fluorophore which is quantified using a fluorescence microplate reader (Molecular Devices SpectraMax iD3). The MCA peptide substrate contains a highly fluorescent 7-methoxycoumarin group that is efficiently quenched by resonance energy transfer to the 2,4-dinitrophenyl group as part of the intact peptide and released after cleavage.

FcγR and Neonatal Receptor (FcRn) Binding

Binding assessment was performed in 1X kinetics binding buffer (ForteBio cat# 18-1105) using an OctetRed96 Biolayer Interferometry instrument. G921 concentrations used were 200, 100, 50, 25, 12.5, and 6.25 µg/mL. Commercial recombinant His tagged receptors are loaded onto anti-His sensors from ForteBio (HIS1K cat# 18-5121) at 5 µg/mL in 1X kinetics buffer for 300 sec and transferred to buffer for baseline measurement (60s). On rate is measured for 300s after transfer of sensor tip to kinetics buffer containing ligand. Off rate is measured for 600s by transfer of sensor tip to kinetics buffer. Recombinant receptors tested are from R&D System (CA), FcγRI (R&D cat # 1257-Fc-050), human FcγRIIa (Cat# R&D-1330), human FcγRIIb (Cat# R&D-1875), human FcγRIIIa (R&D System cat#8894-Fc). For FcRn binding analysis, human recombinant FcRn (R&D System cat#8639-Fc) was used. The sensor tip is loaded with FcRn protein at pH 7.4 followed by baseline at pH 6.0, association with protein at pH 6.0 and dissociation at pH 6.0.

Efficacy Testing in Influenza virus model

Mice were lightly anesthetized by isoflurane. For intranasal inoculation of influenza virus, A/PR/8/34 H1N1, 50 µL of the dose 6.1 Lg EID_{50} was instilled into the nares in one bolus (25 µL/nare) on Day 0. Mice were then moved to their home cage to recover until fully awake. Sham mice were given saline intranasally.

Test article or vehicle at the same volume was administrated on Day 3 via IV route (40mg/kg). A daily maintenance dose was delivered via SC route on Days 4-8 (20mg/kg). Lungs harvested on day 7 for groups to be analyzed by histology. On Day 7, lungs from the vehicle and G921 treated groups were fixed in 10% formalin, embedded, and stained for H&E. The inflamed fraction of diseased lung in affected animals was scored as described.
